# Effect of Mineral Aggregates and Chemical Admixtures as Internal Curing Agents on the Mechanical Properties and Durability of High-Performance Concrete

**DOI:** 10.3390/ma13092090

**Published:** 2020-05-01

**Authors:** Francisco Javier Vázquez-Rodríguez, Nora Elizondo-Villareal, Luz Hypatia Verástegui, Ana Maria Arato Tovar, Jesus Fernando López-Perales, José Eulalio Contreras de León, Cristian Gómez-Rodríguez, Daniel Fernández-González, Luis Felipe Verdeja, Linda Viviana García-Quiñonez, Edén Amaral Rodríguez Castellanos

**Affiliations:** 1 Facultad de Arquitectura (FARQ), Universidad Autónoma de Nuevo León (UANL), 66450 San Nicolás de los Garza, N.L., Mexico; fcofimeuanl@gmail.com; 2 Facultad de Físico Matemáticas (FFM), Universidad Autónoma de Nuevo León (UANL), 66450 San Nicolás de los Garza, N.L., Mexico; nelizond@yahoo.com (N.E.-V.); luz.verastegui.dmz@hotmail.com (L.H.V.); 3 Facultad de Ingeniería Mecánica y Eléctrica (FIME), Universidad Autónoma de Nuevo León (UANL), 66450 San Nicolás de los Garza, N.L., Mexico; amarato2001@yahoo.com.mx (A.M.A.T.); lopez_perales_jesus@hotmail.com (J.F.L.-P.); jose.contrerasle@uanl.edu.mx (J.E.C.d.L.); cristiang1983@hotmail.com (C.G.-R.); 4 Department of Materials Science and Metallurgical Engineering, School of Mines, Energy and Materials, University of Oviedo, Oviedo/Uviéu, 33004 Asturias, Spain; nicadapa@hotmail.com (D.F.-G.); lfv@uniovi.es (L.F.V.); 5 Conacyt-Centro de Investigación Científica y de Educación Superior de Ensenada, Baja California (CICESE UFM), 66600 Monterrey, Mexico; linda@cicese.mx

**Keywords:** concrete, strength, pumice, aggregates, clays, fly ash, limestone, cement

## Abstract

In the present work, the effect of mineral aggregates (pumice stone and expanded clay aggregates) and chemical admixtures (superplasticizers and shrinkage reducing additives) as an alternative internal curing technique was investigated, to improve the properties of high-performance concrete. In the fresh and hardened state, concretes with partial replacements of Portland cement (CPC30R and OPC40C) by pulverized fly ash in combination with the addition of mineral aggregates and chemical admixtures were studied. The physical, mechanical, and durability properties in terms of slump, density, porosity, compressive strength, and permeability to chloride ions were respectively determined. The microstructural analysis was carried out by scanning electronic microscopy. The results highlight the effect of the addition of expanded clay aggregate on the internal curing of the concrete, which allowed developing the maximum compressive strength at 28 days (61 MPa). Meanwhile, the replacement of fine aggregate by 20% of pumice stone allowed developing the maximum compressive strength (52 MPa) in an OPC-based concrete at 180 days. The effectiveness of internal curing to develop higher strength is attributed to control in the porosity and a high water release at a later age. Finally, the lowest permeability value at 90 days (945 C) was found by the substitutions of fine aggregate by 20% of pumice stone saturated with shrinkage reducing admixture into pores and OPC40C by 15% of pulverized fly ash. It might be due to impeded diffusion of chloride ions into cement paste in the vicinity of pulverized fly ash, where the pozzolanic reaction has occurred. The proposed internal curing technology can be considered a real alternative to achieve the expected performance of a high-performance concrete since a concrete with a compressive strength range from 45 to 67 MPa, density range from 2130 to 2310 kg/m^3^, and exceptional durability (< 2000 C) was effectively developed.

## 1. Introduction

Nowadays, reinforced concrete structures are susceptible to chemical attack caused by some elements from the environment. These elements mainly include chloride ions and sulfates. For years, concrete specialists have been trying to increase the service life of the concrete, avoiding the penetration of these harmful agents. To accomplish this tough task, many researchers have focused their efforts on the development of less permeable concrete with low water/cement ratios. In this regard, the use not only of certain chemical admixtures but also supplementary cementitious materials (SCMs) such as pulverized fly ash (PFA) as a synthetic pozzolanic material has contributed to the development of a denser cementitious matrix; thus a less permeable concrete by porosity reduction. The substantial improvement in the properties of the concretes due to the particular addition of chemical admixtures and SCMs have made the concrete, being classified as high-performance concrete (HPC) [1].

Thanks to the high strength-durability performance on structural service under harsh conditions, HPC has been extensively used and investigated around the world [2,3,4,5]. Nevertheless, HPC is characterized by being susceptible to early-age cracking caused by autogenous desiccation and shrinkage [6]. The common definition of HPC refers to concrete mixtures developed due to the concern of durability issues [7]. These concretes are fabricated using low water/cementitious materials (w/cm) ratios, chemical admixtures, and supplementary cementitious materials [8]. However, to achieve durable HPC, the cementitious system has to be well hydrated, meaning that it must be well-cured [9]. Appropriate curing is crucial to the performance of this kind of concrete. Therefore, the improvement in terms of durability; i.e., the decrease in permeability through the use of SCMs, will only be achieved by the development of a well-cured system [10]. HPC with low w/cm ratios (< 0.42) does not have a sufficient amount of water to maintain an adequate saturation state of capillary pores, which would prevent the self-desiccation phenomenon caused by an internal drying of the concrete during hydration reaction. As the cement in a concrete mixture hydrates, water in the capillary pores is consumed, decreasing relative humidity in the mixture and increasing internal stresses; i.e., an increased risk of drying shrinkage and cracking [11,12]. To reduce this risk, it is necessary to mitigate the decrease in relative humidity in the mixture during hydration [10]. In this regard, internal curing (IC) can be helpful to accomplish this task. IC reduces the risk of cracking by the administration of additional water to a concrete mixture to extend the hydration of the cement but without affecting the w/cm ratio [13,14]. Although at the end of the 1950s the benefit of the use of IC was identified, it was not until the 1990s when reemerges, the concept of internal curing in saturated lightweight fine aggregate (LWA) in the concrete mixture to recover the water depleted during cement hydration [15,16,17,18].

For years, several internal curing methods have been proposed to mitigate autogenous shrinkage and the internal stress that might be induced in HPC [3,5,19]. Materials that have been explored as internal curing include: lightweight aggregate (LWA) [20], super absorbent polymer (SAP) [21], perlite [22], bentonite clay [23], rice husk ash (RHA) [24], coal bottom ash [25], cenospheres [26] and wood pulp [11], among others.

Internal curing materials can be classified into two categories following the water-absorbing mechanism: substances containing physically adsorbed water and porous materials [19]. At the same time, porous materials can be classified into LWA and porous superfine powders [19]. LWA materials include leca (expanded clay) [12,18,27,28,29,30,31,32,33,34], pumice stone (PS) [35,36], zeolite [27,37], perlite [22], and bio-LWA [38]. On the other hand, porous superfine powders include RHA [39,40], acid leaching residue of boron mud [41], and bottom ash [25].

LWA has been used not only as an internal water source but also as a medium to dose additive mixtures within the concrete without affecting the properties of concretes in the fresh state. Besides, two characteristics in LWA are currently being studied: water absorption and desorption capacity. Recent studies have indicated that the use of large amounts of the LWA eliminates autogenous shrinkage in the concrete. Nonetheless, this implementation causes a decrease in compressive strength. Therefore, some studies have been conducted to evaluate the effect of using a few amounts of lightweight aggregate with an optimum particle size on the cementitious matrix [35].

The technology of porous saturated aggregates with additives consists of the use of solutions introduced as an internal curing medium to increase the durability of the concrete with a higher viscosity than water. The physical principle is based on the Stoke-Einstein equation (Equation (1)):

(1) $$D_{0}=\frac{k_{B}T}{6\pi \eta r}$$

where $D_{0}\;$ is the proper diffusion coefficient of an ion, $k_{B}$ is the Boltzmann constant, T is the temperature, r is the radius of the particle that diffuses, and η is the viscosity of the particle that diffuses [1,24,42,43].

The IC method using LWA consists in using the same size and fractions as a normal fine limestone aggregate to provide the most efficient supply of internal curing water to the mixtures so that it is covered most of the sites in the paste volume at a maximum level.

Although a suitable amount of water is supplied by the saturated LWA, it must be well distributed in the mixture to moisture all of the paste. The required spacing called “the spacing factor” is described as the proximity of the LWA with the paste inside the concrete matrix. This spacing factor has been studied since it influences the internal water desorption. As known, the autogenous shrinkage is in the function of the spacing between LWA and the paste [44].

Moreover, the PS as a curing agent facilitates the internal curing process due to its high porosity and thick porous structure since it allows the absorption of fluids when it is submerged in aqueous solutions. Besides, this aggregate can desorb the solution inside the concrete causing high relative humidity. The PS morphology makes it possible that the water can lodge in the capillary pores of the aggregate. The optimum pore size for storing the internal curing water is approximately 100 nm. If the pores are smaller than 100 nm, the water remains tightly bonded, and it is not available for cement hydration reactions in the hardening process [39].

On the other hand, one of the materials with the highest compressive strength among lightweight aggregates is expanded clay aggregates (leca). Leca is an artificial and manufactured lightweight aggregate. After heating at 1150 °C in a rotary kiln, the clay expanded to about four to five times its original size and took the shape of pellets [33]. Then, this expansion becomes porosity in the aggregate. This porosity is beneficial due to a density reduction in the aggregate as well as it can be used to store moisture for the internally cured concrete [3]. Besides, it is also possible to store some other solutions as shrinkage reducing admixtures or corrosion inhibitors [45]. Some studies have shown that expanded clays (EC) permit an absorption between 15% and 31% during the first 24 h [29,30]. Furthermore, EC can desorb between 85% and 98% of the water absorbed during its first 24 h (at 93% relative humidity). These two features of the EC improve the performance of internal curing, producing concrete possessing compressive strength as high as 80 MPa [29,30,31,32,33,34]. This is particularly possible due to the thickness and the quality of the ITZ of expanded clay aggregate since are superior to normal aggregate concrete [33].

Following the above mentioned, it is well recognized that the internal curing technique of presoaked-lightweight aggregates improves the properties of the concrete since it has been proven that the autogenous shrinkage of high-performance concretes decreases with the increase of the induced internal curing water. However, internal curing cannot eliminate the retractions of high-performance concrete [46,47,48,49]. Consequently, the use of shrinkage reducing additives (SRA) in this kind of concrete is needed to decrease plastic shrinkage and cracking when exposed to room temperature. Based on the patent of Goto et al. [50], SRA is a class of organic chemicals known as surfactants [51] composed of a hydrophilic (i.e., polar) head that is covalently bonded to a hydrophobic (i.e., non-polar) tail. Many studies agree with the conceptualization that SRA decreases the evaporation of water in pore fluids (i.e., less settlement) and reduces capillary tension (i.e., fewer cracks formation). Therefore, it is widely accepted that SRA can reduce the shrinkage of cementitious materials [51,52,53,54,55,56].

Recently, new techniques have evolved to improve the physical-mechanical performance and durability of structural concrete. One of these techniques includes the combination of mineral aggregates with chemical admixtures to improve high-performance concrete durability since might help to mitigate the diffusion of chloride ions and the penetration of accelerated carbonation [57,58,59,60].

In particular, the resistance to chloride ions penetration is an important matter in concretes applied in marine construction, where there is a high risk of steel corrosion reinforcement concrete induced by chloride [61,62,63,64,65,66]. Experimental results have shown that the chloride diffusivities of high- performance concrete with lightweight aggregate were similar or, in some cases, lower compared to that normal aggregate concrete [61,67]. This phenomenon is attributed to the dense interfacial transition zone (ITZ) between the lightweight aggregates and cement paste, which would hinder the penetration of chloride ions [61,68,69].

Since the principle and design method of the combined use of mineral aggregates with chemical admixtures are still unclear, the goal of this study is to evaluate the effect of mineral aggregates pumice stone and expanded clay aggregate along to the chemical admixtures superplasticizers and shrinkage reducing additive as internal curing agents to improve the physical, mechanical, and durability properties of high-performance concretes.

## 2. Experimental Procedure

### 2.1. Materials

The raw materials used to elaborate the concretes are commercially available products, such as a CPC30R (Portland composite cement) and OPC40C (ordinary Portland cement) from CEMEX (Monterrey, Mexico), coarse and fine aggregates from limestone minerals (from here identified as CA and FA, respectively), pulverized fly ash (PFA), pumice stone (PS), and clay aggregates for curing. This clay aggregate is also known as expanded clay (EC).

Tetraguard AS20 shrinkage reducing admixture from BASF (Ludwigshafen, Germany) was the commercial chemical admixture used in this research to reduce drying shrinkage of concrete and the potential for subsequent cracking. From here this chemical admixture is referred to as SRA.

Glenium 3030 NS superplasticizer from BASF was used to produce concrete with high flowability. Glenium 3030 is an admixture based on polycarboxylate chemistry. The dosage is for each cement kilogram, 1% of superplasticizer was added for internal curing concrete.

Table 1 collects the chemical composition (according to the oxide content) of the raw materials analyzed by X EPSILON 3-XL X-ray fluorescence equipment (Malvern Panalytical Ltd, Malvern, Worcs, UK).

The chemical analyses results showed a high concentration of calcium in the mineralogical compounds of the ordinary Portland cement. Thus, the calcium concentration is quite enough to generate gels of hydrated calcium silicate with a stoichiometric Ca/Si ratio higher than 3.2 in the hydration process. Additionally, it is important to consider the content of alkalis (0.6 wt.% of Na_2_O and 0.1 wt.% of K_2_O) since it could be carried out an alkali-aggregate reaction.

The physical properties of the aggregates used in the present work are shown in Table 2. These physical properties (density, blaine, absorption, and volumetric weight) were evaluated by the following ASTM standards: ASTM C29-17 [71] (Standard Test Method for Bulk Density (“Unit Weight”) and Voids in Aggregate), ASTM C204-17 [72] (Standard Test Methods for Fineness of Hydraulic Cement by Air-Permeability Apparatus), and ASTM C128-15 [73] (Standard Test Method for Relative Density (Specific Gravity) and Absorption of Fine Aggregate). The ASTM C138-17 [74] Standard Test Method for Density (Unit Weight), Yield, and Air Content (Gravimetric) of Concrete) was used for the concrete elaboration to structural use with resistances greater than 30 MPa at 28 days, as specified in the ACI 318 (American Concrete Institute, Farmington hill, MI, USA).

**Figure 1:**
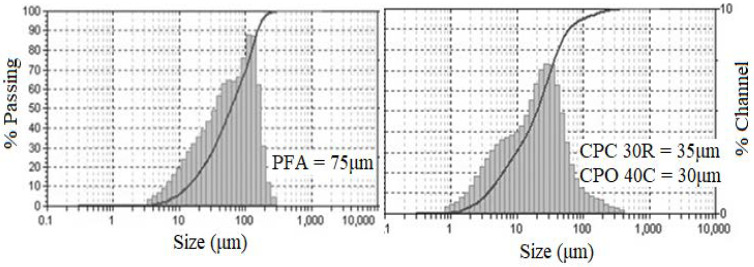
The average particle size of cementitious materials.

The cementitious powders were sieved by a laser granulometry technique in a particle size analyzer model MICROTAC 3500 (Microtract, Montgomeryville, PA, USA). Figure 1 shows the average particle size for the CPC30R (35 μm) and the OPC40C (30 μm). Besides, the PFA particle size was found in the range of 40 μm to 110 μm with an average particle size of 75 μm. It is expected that the PFA would improve the properties of the cementitious matrix since the size of its particles, is twice in size compared to Portland cement particle size. Therefore, PFA particles can be acting as filler.

As known, it is important to determine the morphology, pore size, and other characteristics of the aggregate since its capacity as an internal curing medium to increase the durability of the concrete depends on these factors. The microstructure of the PS aggregate was analyzed by scanning electron microscopy. The samples were coated with a thin layer of graphite to make the sample conductive as well as to obtain a better resolution in the scanning electron microscope (SEM). The microstructure analysis was performed on a JSM-6490LV electronic microscope (JEOL, Akishima, Tokyo, Japan) coupled with an INCA-Sight energy dispersive X-ray spectrometer (EDX) from Oxford Instruments (Akishama, Tokyo, Japan).

**Figure 2:**
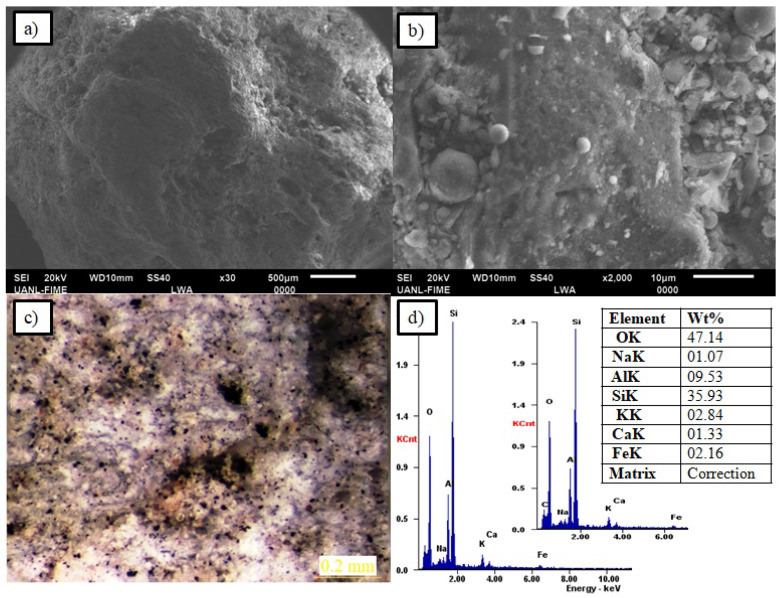
Pumice stone characterization. (**a**) Morphology of the PS aggregate by SEM; (**b**) Internal structure of the PS aggregate by SEM; (**c**) Pore distribution in a PS particle by stereoscope; (**d**) Silica-alumina concentration by EDX analysis.

Figure 2 shows the microstructural characteristics of PS aggregates. By the SEM analysis, it was shown that the PS aggregate has a high porosity, which represents a desirable characteristic for the curing process since the aggregate absorbs the aqueous solution and the additional water as an internal curing agent. The pore size of the PS aggregate ranges from 100 nm to 10 μm. This pore size range is beneficial since the aggregates with pore size below 100 nm tend to fail when releasing the internal water due to the surface tension of water. Figure 2a shows a general view of the exterior morphology of the PS aggregate. In Figure 2b, the PS internal structure is shown. As a complement, Figure 2c shows the pore distribution through the entire PS particle using a stereoscope. The EDX analysis carried out in the PS particle is shown in Figure 2d, where it can be observed the concentration of siliceous and aluminum forming mainly a silico-aluminous compound.

### 2.2. Concrete Mixtures

Concrete mixtures were made in two stages. Stage 1 is derived from the results obtained by Trujillo et al. [60], where four different types of internal curing concretes were designed to counteract the autogenous shrinkage phenomenon. In stage 1, the control concrete was elaborated with 100% of CPC30R cement and none internal curing method was used. These four internal curing concretes were made with replacement of normal fine aggregates for pumice stone and clay aggregates in 20% of the mass along with the replacement of PFA in 15% for the Portland cement compound (CPC30R). In stage 2, four types of internal curing concretes using ordinary Portland cement (OPC40C) were designed, according to the ASTM C 150-18 [75] (Standard Specification for Portland cement). In stage 2, these four internal curing concretes were made with replacement of normal fine aggregates for pumice stone in 20% of the mass along with the replacement of PFA in 15% for the ordinary Portland cement (OPC40C). In stage 2, the control concrete was elaborated with 100% of OPC40C cement and none internal curing method was used.

Table 3 shows the codes and constituents of the experimental concrete mixtures made with CPC30R and OPC40C. All internal curing mixtures contain a superplasticizer additive and a shrinkage reducing admixture to improve the physical properties of concretes. Table 4 and Table 5 show the percentage of the constituent of the concrete elaborated in stages 1 and 2, respectively.

### 2.3. Specimen Preparation

All concrete mixtures were prepared in a laboratory mixer using the same mixing procedure. For each type of concrete, all the specimens were prepared from the same batch.

#### 2.3.1. Curing conditions in stage 1

At a fresh and hardened state, the specimens were kept in the molds under laboratory conditions in a controlled climate chamber at a temperature of 25 ± 1 °C and 60 ± 2% of relative humidity (RH). At the hardened stage, the specimens were kept in their molds for 24 h and protected from moisture exchange. Then the specimens were subsequently unmolded and cured according to the specifications of the ASTM C 531-00 [76] (Standard Test Method for Linear Shrinkage and Coefficient of Thermal Expansion of Chemical-Resistant Mortars). Finally, the specimens were aged for 180 days [60].

#### 2.3.2. Curing conditions in stage 2

At the fresh and hardened state, the specimens were kept in the molds under laboratory conditions in a controlled climate chamber at a temperature of 25 ± 1 °C and 60 ± 2% of relative humidity. The specimens were cured by a submerging procedure for 14 days as specified in the ASTM C31-18 [77] Standard Practice for Making and Curing Concrete Test Specimens in the Field).

### 2.4. Testing Methods

Concrete in the fresh state (concrete mix testing):

The properties of fresh concrete were immediately determined after mixing sequence and before concrete specimens casting. The concrete mixing was evaluated as specified by the ASTM C94-17 [78] (Standard Specification for Ready-Mixed Concrete).

#### 2.4.1. Volumetric Weight

The volumetric weight was evaluated as specified by the ASTM C138-17 [74] (Standard Test Method for Density (Unit Weight), Yield, and Air Content (Gravimetric) of Concrete).

#### 2.4.2. Air Content

The air content was performed by the volumetric method specified by the ASTM C173-16 [79] (Standard Test Method for Air Content of Freshly Mixed Concrete by the Volumetric Method).

#### 2.4.3. Slump

The slump was evaluated as specified in the ASTM C143-15 [80] (Standard Test Method for Slump of Hydraulic-Cement Concrete).

Concrete in the hardened state.

#### 2.4.4. Compressive Strength

**Figure 3:**
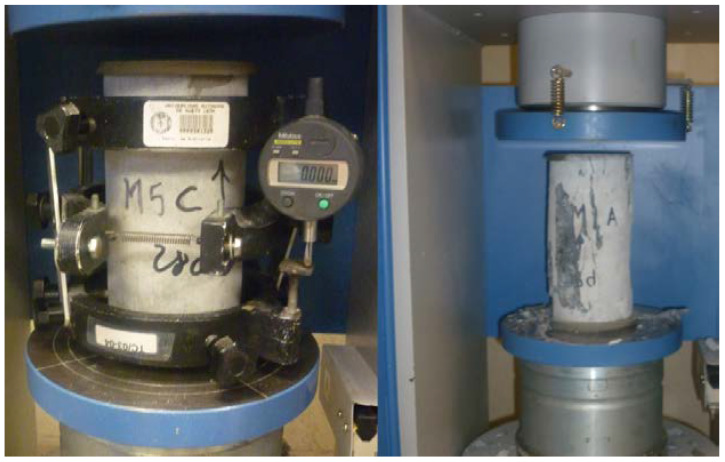
Failure of the concrete sample in the compressive test.

The specimens were mechanically evaluated using a mechanical testing machine trademark ELE at a loading speed of 3.5 kg/cm^2^ per second, according to the procedures described by the ASTM C39-18 [81] (Standard Test Method for Compressive Strength of Cylindrical Concrete Specimens). Figure 3 shows the compressive test for these concretes. The compressive strength was measured on five cylindrical samples measuring 100 mm × 200 mm. All strength data represents an average of five specimens.

#### 2.4.5. Microstructural Evaluation

Experimental concretes were microstructurally analyzed by SEM-EDX techniques to study the ITZ and its effect on the densification of the cementitious matrix. A JEOL JSM-6490LV scanning electronic microscopy coupled with an energy dispersive X-ray spectrometer (EDX) INCA-Sight from OXFORD was used by the microstructural analysis. This was already stated a few pages back – remove one.

#### 2.4.6. Open Porosity

The open porosity of hardened concrete at 28, 90, and 180 days of curing were measured with 100 × 50 ± 5 mm cylindrical specimens following the test procedure in the ASTM C642-13 [82] (Standard Test Method for Density, Absorption, and Voids in Hardened Concrete, for their subsequent analysis). The open porosity was measured on five cylindrical samples.

#### 2.4.7. Depth of Chloride Ion Penetration

The chloride permeability evaluation on the concretes was determined using Nordtest NT BUILD 443 equipment (Nordic innovation, Oslo, Norway) according to the ASTM C1202-12 [83]. Based on the compositions given in Table 3, cylindrical samples of 100 mm in diameter were processed and then cut into sections of 50 mm in thickness. A setting epoxy resin was used to coat the cut sections that will be put into voltage cells to calculate the electric charge passed through them. A 3 % NaCl solution was used to connect the cathodic area and a 0.3 M NaOH solution to connect the anodic area. Finally, a 60 VDC potential difference was applied during the measurement between the two sections for 6 h. The voltage and resistance between the cells were recorded every 30 min until complete the 6 h. The current that passes through the cell was calculated from the voltage measurements, and the total passed charge was calculated using Equation (2) as:

(2) $$Q=900\times \left[I_{0}+2I_{30}+2I_{60}+\ldots +2I_{300}+I_{360}\right]$$

The total charge passed through the sliced specimen in coulombs (C) is referred to Q and the current (A) at n minutes after the voltage is applied as I. When the Q value is less than 2000 C is indicated as low chloride permeability, in a value range from 2000 C to 4000 C, the chloride permeability is at a medium level, and Q values higher than 4000 C are established as high chloride permeability. After the test was finished, the penetration depth was measured. A 0.1 M AgNO_3_ solution was sprayed onto the fracture surfaces of sliced samples that were split into two sections. The penetrated zone was determined by the change in color on the fracture surfaces to a brownish tone. For each concrete composition, the total charge was measured two times, and the penetration depth from 10 measuring points on each fracture surface.

## 3. Results and Discussion

### 3.1. Properties of Concrete in the Fresh State

The volumetric weight and the air content in the experimental concrete provide information on the workability, trapped air, and porosity that the concrete may develop in the hardened state. The results show that the concrete elaborated in stage 1 with clay aggregate, registered a volumetric weight ranging from 2100 kg/m^3^ to 2400 kg/m^3^ [60]. Moreover, the volumetric weight in the concrete of stage 2 ranges from 2260 kg/m^3^ to 2400 kg/m^3^. Table 6 shows the volumetric weight values obtained in the experimental concretes of this study, which is in the established range to be classified as structural concrete according to the ASTM C138-17 [74] (Standard Test Method for Density (Unit Weight), Yield, and Air Content (Gravimetric) of Concrete). The R1 mixture showed a change in volumetric weight around 2.12% more than the R mixture. The RFA and IC2 mixtures presented the same volumetric weight values (2310 kg/m^3^). The ICSRA mixture increased its volumetric weight by 8.5% concerning to the IC mixture. It should be mentioned that the volumetric weight in the concrete, where fine aggregate was substituted by the internal curing agents (clay aggregate and pumice stone), only varied approximately 5%.

Table 7 shows the air content values into the concretes in a fresh state. The highest percentage of air entrapment (4%) was observed in the concrete made with the pumice stone. This phenomenon can be attributed to the high porosity of PS, as was corroborated through the SEM analysis. It should be mentioned that the workability of the concretes was good enough since the slump values were found higher than 13 cm. Besides, the mixtures with the addition of the PFA reached slump values up to 21 cm. This can be attributed both to the lower consumption of cement and a large amount of desorption water from lightweight aggregates (clay aggregate and pumice stone).

### 3.2. Properties of Concrete in the Hardened State (Compressive Strength)

**Figure 4:**
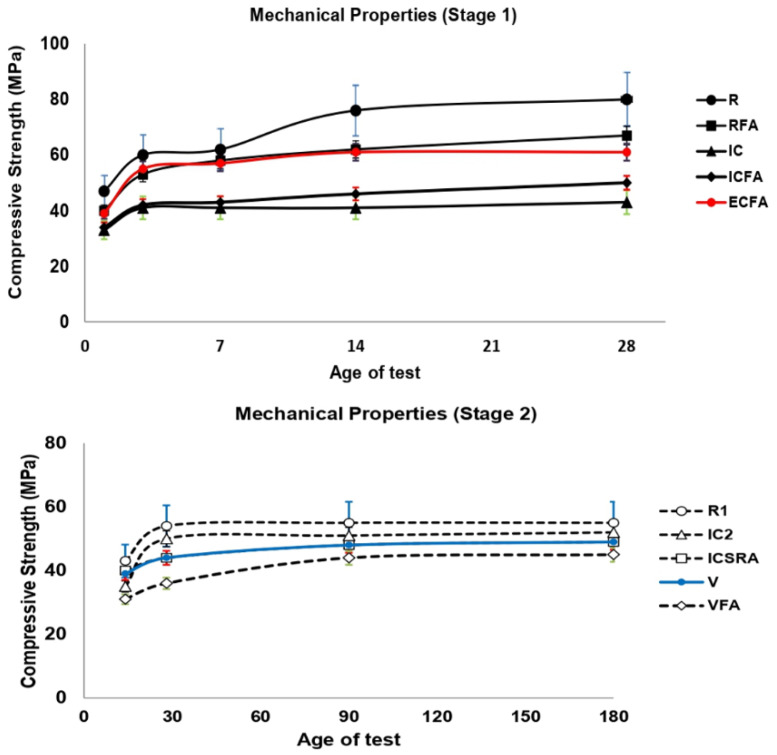
The compressive strength of concretes in stages 1 and 2.

Figure 4 shows the compressive strength results evaluated at 1, 3, 7, 14, and 28 days for all concretes corresponding to stage 1 and 14, 28, 90, and 180 days for all concretes corresponding to stage 2.

As can be observed, at stage 1, the control concrete (R) has a drastic increase in compressive strength from 45 MPa (during the first curing day) to 80 MPa (after being evaluated at 28 curing days). This strength improvement leads to an increase of about 78% from the initial age (1 day) to the final age (28 days). As expected, the pozzolanic additions from CPC30R contained in the reference concrete (R) lead to the formation of carboaluminates and carbosilicates, which directly influenced to obtain the highest mechanical resistance of all the studied concrete [85,86]. Besides, the lack of an internal curing and a weak porous medium contributes to reaching this highest strength. In RFA concrete, where CPC30R is partially replaced by 15% PFA, the maximum compressive strength of 67 MPa was observed at 28 days. The 16% loss in mechanical strength of RFA concrete compared to reference concrete at this age, can be mainly attributed to less calcium silicate hydrate (CSH) gel formation due to the reduction of Portland cement in the concrete. Among the concretes made with the addition of an internal curing agent (IC, ICFA, and ECFA), the concrete containing aggregates of expanded clay as a curing agent (ECFA) showed the highest compressive strength (61 MPa) at 28 days. Although the compressive strength of ECFA concrete is lower than that of reference concrete (29%), the strength is higher than those reached by the rest of the concretes that contain an internal curing agent (ICFA and IC). In this sense, the higher strength of ECFA concrete is attributed to the use of a less porous curing agent (expanded clay) compared to pumice stone, which allows developing a denser matrix with higher strength [32]. Even though the standard dictates a minimum compressive strength of 25 MPa at 28 days, the compressive strength values of the proposed concrete exceed 30 MPa at the initial age (1 day), which indicates that the minimum strength is reached at early age.

Following a similar behavior, the compressive strength obtained in all concretes tested in stage 2 exceeds the compressive strength of 30 MPa at the first day of age; i.e., it was accomplished the minimum compressive strength indicated by the standard (25 MPa at 28 days) at an earlier age. Then, at 90 days of age, the variation in compressive strength in all concretes evaluated was negligible. In this stage, the highest compressive strength was reached by the R1 concrete (55 MPa at 90 days). Likewise, the lack of an internal curing agent and weak porous medium contributes to achieving this highest strength. Furthermore, in IC2 concrete, which PS replaced 20% of fine aggregate (FA), compressive resistance of 35 and 52 MPa at 14 and 180 days, respectively, was reached. Among the concretes made with the addition of an internal curing agent (V, ICSRA, and VFA), the concrete IC2 showed the highest compressive strength at 180 days. A loss of 7% in the strength of IC2 concrete compared to the R1 concrete strength at 28 days can be mainly attributed to a higher porosity in the matrix due to the use of a highly porous curing agent (pumice stone); i.e., a weaker matrix that develops lower strength. In IC2 concrete, it is worth noting the increase in compressive strength from 35 MPa at 14 days to 50 MPa at 28 days. The 42% increase in mechanical resistance indicates that the expected effect of the internal curing agent is being achieved, mainly due to a significant release of water at later ages. The V and ICRSA concretes obtained a compressive strength of 49 MPa at 180 days, which represents an 11% reduction in mechanical resistance compared to the reference concrete. In both, 20% of fine aggregates (FA) were replaced by pumice stone (PS), and a shrinkage reducing admixture was added. The addition of PS in the V concrete was made after the saturation of PS by its immersion into a solution of water with the SRA while in the ICSRA concrete, the PS was saturated in water and then was added into the mixture with the SRA together with the others concrete components. Although different methods were used to add the PS to the mix, the mechanical resistance of both concretes remained practically constant at all ages. In the VFA concrete, where 20% of fine aggregate (FA) were replaced by pumice stone (PS) saturated with shrinkage reducing admixture into pores in addition to 15% of OPC40C by PFA, the lowest compressive strengths were reached with 31 and 45 MPa at 1 and 180 days, respectively. Moreover, VFA concrete developed the lowest compressive strength at 180 days compared to all concretes studied. The average reduction in strength was: R1 concrete (~ 18%), IC2 (~ 13%), ICSRA and V (~ 8%). Certainly, the diminishing in cement concentration by its substitution for PFA in the VFA concrete is the main reason for the strength decrease.

As expected, R concrete developed higher compressive strength (80 MPa) at 28 days than R1 concrete (54 MPa). In CPC30R, the pozzolanic was relevant for developing higher mechanical resistance in concrete R (~ 32%). On the other hand, IC2 concrete showed higher compressive strength compared to IC concrete at 28 days (~ 14%). This is due to a better interaction of the PS aggregate in the OPC for IC2 concrete, unlike the PS aggregate in the CPC for concrete IC. This phenomenon can be explained due to the combined effect of desorption of water from the light aggregate and the action of the superplasticizer admixture. The latter causes steric repulsion in the Portland cement particles, which in turn results in hydration of more cement particles, and therefore a higher amount of CSH gel is formed. Internal curing concretes with PS additions based on OPC (IC2, V, and ICSRA), showed superior strength at 28 days than that based on CPC (IC concrete). In the same sense, ICFA concrete showed higher compressive strength (50 MPa) than VFA concrete (36 MPa) at 28 days. This higher strength represents an improvement of 28%. The lack of OPC and CPC by its substitution for PFA affects the compressive strength in both concrete. However, the ICFA strength is less affected than the VFA strength since PFA in the ICFA concrete is acting as a filler that contributes to higher strength. Finally, the concrete with expanded clay addition (ECFA concrete) experiments higher strength (61 MPa) than ICFA, and VFA concretes with the addition of pumice stone (50 and 36 MPa) at 28 days. The higher resistance is attributed to the fact that the expanded clay is a less porous curing agent than pumice stone, which led to a denser matrix with higher strength. The higher strength of the ECFA concrete versus ICFA and VFA concretes strength represents an improvement of 18% and 41% respectively.

### 3.3. Microstructural Evaluation

**Figure 5:**
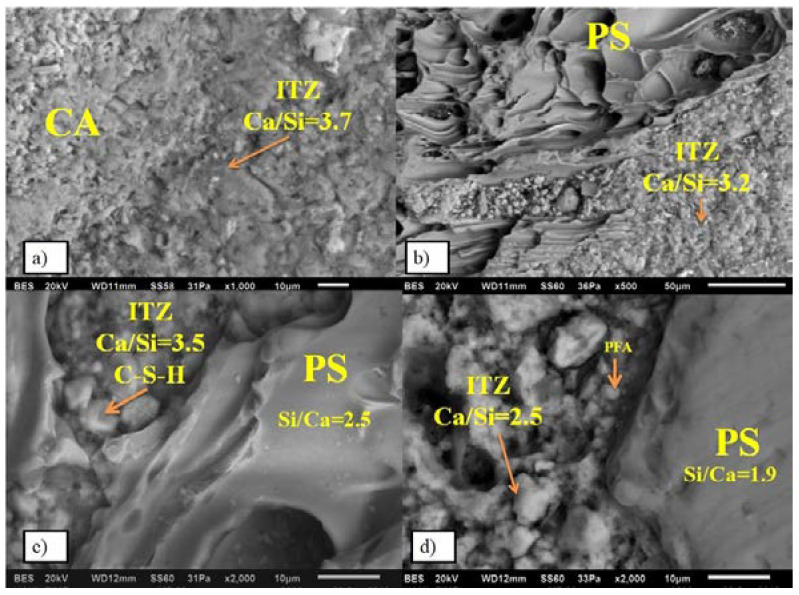
Microstructural evaluation through the Ca/Si ratio in ITZ. (**a**) ITZ between coarse aggregate and cementitious matrix in R1 concrete; (**b**) ITZ between pumice stone and cementitious matriz in R1 concrete; (**c**) ITZ between coarse aggregate and cementitious matrix in ICSRA concrete; (**d**) ITZ between PS and cementitious matrix in ICSRA concrete.

Figure 5a shows the microstructure of R1 concrete. It is observed that between the coarse aggregate identified as CA and the cementitious matrix, there is a Ca/Si ratio of 3.7. This value represents a high calcium concentration leading to the formation of long CSH gel chains, which allows high densification of the matrix. Figure 5b shows the interface between the PS and the cementitious matrix. It can be observed that the Ca/Si ratio remained at the stoichiometric value (3.2). Figure 5c shows the ITZ of the ICSRA concrete. Here, the Ca/Si ratio evaluated by EDX analysis was 3.5 in the ITZ, while the Ca/Si ratio was 2.5 in the PS zone. Figure 5d shows the ITZ between the PS and the cementitious matrix. It can be observed a Ca/Si ratio of 2.5. It is due to the interaction of the CSH gels with the pozzolanic reaction of fly ash along with a lower cement addition.

A representative specimen from the ICSRA concrete was taken to be analyzed in a stereoscope to determine the macroscopic effect of the pumice stone in the concrete matrix. A brighter coloration in the periphery area of pumice stone is observed, mainly due to the formation of a CSH-rich gel. This gel is promoted by more exceptional hydration in the region of the aggregate/paste interface. Also, this analysis confirms the homogeneity of all the aggregates in the concrete matrix.

**Figure 6:**
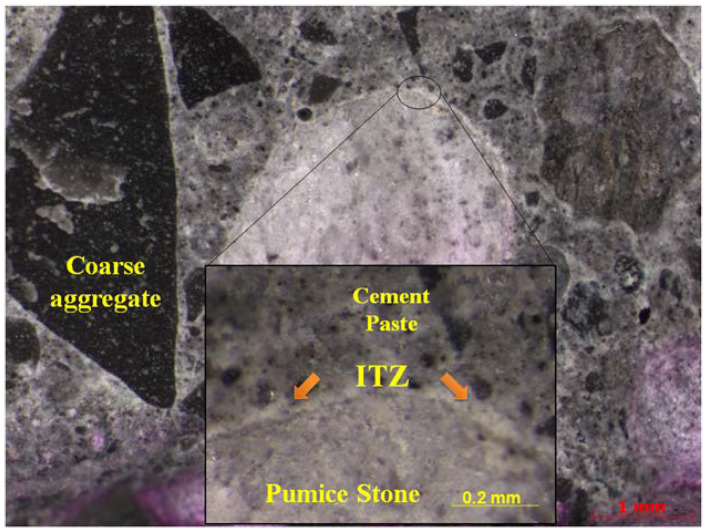
Petrography of the ICSRA concrete.

Figure 6 shows a representative specimen from the ICSRA concrete taken to be analyzed in a stereoscope to determine the macroscopic effect of the pumice stone in the concrete matrix. A brighter coloration in the periphery area of pumice stone is observed, mainly due to the formation of a CSH-rich gel. This gel is promoved by more exceptional hydration in the region of the aggregate/paste interface. Also, this analysis confirms the homogeneity of all the aggregates in the concrete matrix.

### 3.4. Open Porosity

**Figure 7:**
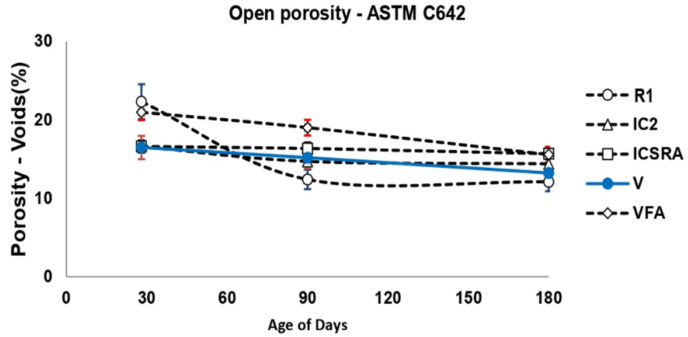
Open porosity of the concretes elaborated in stage 2.

Open porosity represents an important parameter since the behavior of concrete against the diffusion of fluids, whether chlorides or carbonation, can be predicted through the percentage obtained. Figure 7 indicates that R1 and VFA concrete reached a porosity value of 22.3% and 20.96%, respectively at 28 days. Then, at the age of 180 days, the porosity of R1 and VFA concretes decreased by ~ 45% and ~ 26% respectively. On the other hand, IC2, ICSRA, and V concrete with additions of the pumice stone showed almost the same porosity percentage (16%) at 28 days. Finally, at the age of 180 days, the open porosity decreased by 12.78%, 5.542%, and 19.73% for IC2, ICSRA, and V concrete, respectively. As known, the high open porosity and interconnectivity of pores allow the PS to absorb a higher quantity of water and, subsequently, a faster release of it; both factors lead to a denser matrix by a suitable internal-curing.

### 3.5. Penetration Depth of Chloride Ions

**Figure 8:**
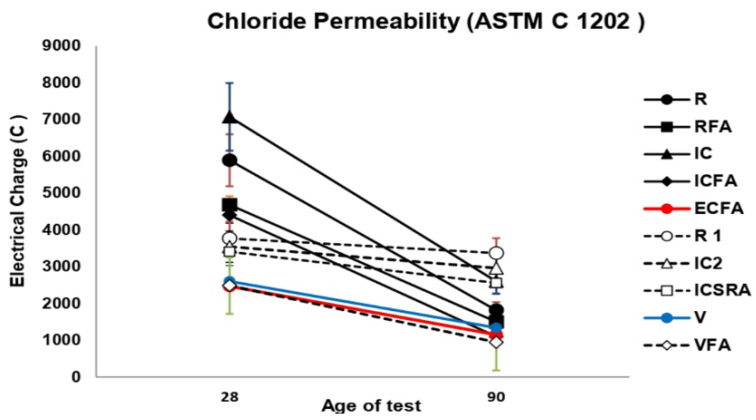
Comparison of the transmitted load of chloride ions in concretes elaborated in stage 2.

Figure 8 shows the chloride penetration results determined in the concretes elaborated in stage 2. The VFA concrete exhibited the lowest permeability value at 90 days (945 C). However, it is necessary to consider that this concrete also showed the highest porosity at 90 and 180 days (~ 19% and ~ 16%). As it is well known, the concrete porosity is strongly related to the chloride penetration in the concrete matrix. Chia and Zhang found that the chloride penetration in concrete was mechanically affected by porosity in the concrete and the connectivity of pores [67]. Therefore, even if the concrete porosity is high, chloride penetration may be low when the connectivity of pores is low. As mentioned above, in the VFA concrete, 20% of fine aggregate (FA) were replaced by pumice stone (PS) saturated with shrinkage reducing admixture into pores in addition to 15% of OPC40C by PFA. So, the low permeability value found in VFA concrete indicates the development of low connectivity between pores into the cement matrix.

Similar behavior was exhibited in terms of permeability at 90 days by ICFA (1100 C) and ECFA (1150 C) concretes. The VFA, ICFA, ECFA, V (1338 C), and RFA (1500 C) concretes exhibited outstanding performance since their permeability values at 90 days are below 1500 C with almost no penetration. As data, except for the V concrete, the latter group of concretes has the replacement of Portland cement by PFA as a feature. This fact suggests that PFA influenced obtaining low pore connectivity in the cementitious matrix.

For concrete V, a reduction in the permeability value from 2600 C (28 days) to 1338 C (90 days), indicates that the internal curing method as a densification medium was effective. R, ICSRA, and IC concrete showed a moderate penetration of chloride ions at 90 days (1815 C, 2556 C, and 2600 C, respectively). As mentioned above, in ICSRA and IC concretes, 20% of fine aggregates (FA) were replaced by pumice stone (PS) and a shrinkage reducing admixture was added. Moreover, based on the permeability reduction from 28 days to 90 days, the IC concrete achieved a greater reduction (63%) than the ICSRA concrete (25%). Therefore, the internal curing method on the IC concrete was more effective than the ICSRA concrete.

Furthermore, even though R concrete did not develop internal curing, it exhibited moderate permeability due to the formation of a dense matrix. Finally, IC2 and R1 concrete showed the highest permeability values at 90 days (2960 C and 3368 C). A high enough connectivity of the pores in the cementitious matrix could be the cause of the permeability values reached in these concretes. In general, it was possible to observe that the total permeability of chloride ions in concretes with pulverized fly ash (PFA) contents and internal curing, was lower compared to the reference concretes (R and R1). It might be due to impeded diffusion of chloride ions into the cement paste in the vicinity of PFA, where the pozzolanic reaction has occurred.

Based on the overall experimental data in the present study, it can be observed that the combined use of pulverized fly ash with curing agents in high-performance concrete can significantly reduce the amount of chloride diffusion.

On the other hand, it is well-accepted that SRA improves the water absorption and chloride permeability of concrete due to the reduced surface tension of pore solution, increased viscosity, and refinement of pore structure. However, in the present study, the effect of SRA merely affected the durability property. The effect of superplasticizer on the durability was not clearly understood.

## 4. Conclusions

High-performance concrete has numerous environmental and societal benefits of structural service under harsh conditions. HPC is characterized by being susceptible to the risk of early-age cracking because of autogenous desiccation and shrinkage. The development of a well-cured system is decisive for high-performance concrete to improve durability. Since the principle and design method of the combined use of mineral aggregates with chemical admixtures are still unclear, the goal of this study was to evaluate the effect of mineral aggregates (pumice stone and clay aggregate) and chemical admixtures (superplasticizers and shrinkage reducing additive) as internal curing agents.

In the present research work, an effective internal curing method consisting of the combined effect of substituting Portland cement for mineral aggregates along with chemical admixtures for the manufacture of high-performance concrete was applied.

Mixtures with partial replacements of CPC30R/OPC40C by pulverized fly ash (PFA) in combination with the addition of pumice stone (PS)/expanded clay (EC) and superplasticizer/shrinkage reducer studied in the fresh and hardened state, allowed to obtain the following conclusions:

(1) Structural high-performance concretes with compressive strengths of 45 to 67 MPa and densities of 2130 to 2310 kg/m^3^ were obtained. All these ranges are favorable for the development of structural concretes. The structural efficiencies of these concretes are much higher than the conventional normal density concretes.
(2) The adverse effect of internal curing on the mechanical strength of the concrete at early-age was improving at later ages, where acceptable compressive strengths were achieved.
(3) A less porous curing agent such as expanded clay (EC) compared to pumice stone (PS) allowed to exhibit the highest mechanical strength at 28 days (61 MPa), due to the development of a denser cementitious matrix (ECFA at stage 1).
(4) In IC2 concrete, where 20% fine aggregate (FA) was replaced by pumice stone (PS), the effect of high water release by the internal curing agent allowed to increase the compressive strength from 35 MPa at 14 days to 50 MPa at 28 days, and subsequently achieve the highest compressive strength among the concretes with an internal curing agent at 180 days.
(5) The VFA concrete exhibited the lowest permeability value at 90 days (945 C). This concrete included substitutions of fine aggregate by 20% of the PS saturated with shrinkage reducing admixture into pores and substitutions of OPC40C by 15% of PFA. Interestingly, the VFA concrete exhibited the highest porosity at 90 days and 180 days (~ 19% and ~ 16% respectively). The relationship between the porosity in the concrete and the connectivity may affect the chloride penetration. Even if the porosity of concrete is high, chloride penetration may be low when the connectivity of pores is low. Therefore, it was presumed that the VFA concrete developed low connectivity between pores into the matrix.
(6) Similar behavior in permeability at 90 days was exhibited by ICFA (1100 C) and ECFA (1150 C), V (1338 C), and RFA (1500 C) concrete. This concrete showed outstanding performance since their permeability values at 90 days are below 1500 C with almost no penetration.
(7) The total of chloride ions permeability in internal curing concrete with pulverized fly ash (PFA) were lower than those in the control concrete (R and R1 concrete). It might be due to impeded diffusion of chloride ions into cement paste in the vicinity of PFA, where the pozzolanic reaction has occurred.
(8) As known, it is generally believed that SRA improves the water absorption and chloride permeability of concrete due to the reduced surface tension of pore solution, increased viscosity, and refinement of pore structure. However, in the present study, the effect of SRA merely affected the durability property.
(9) The effect of superplasticizer on the concrete properties was not clearly understood.

Considering the overall experimental data in the present study, it can be said that the combined use of pulverized fly ash with curing agents in high-performance concrete can significantly reduce the amount of chloride diffusion. Further research will be conducted along the lines of the present study to evaluated the transmittance charge responsible for migration promotion.

## Figures and Tables

**Table 1:** Chemical analysis of the raw material obtained by X-ray fluorescence (wt.%).

Raw Material	CaO	SiO_2_	Al_2_O_3_	Fe_2_O_3_	Na_2_O	K_2_O
PS	2.7	72	143	3.7	1.7	0.1
EC [70]	0.2	58	27	1.0	0.3	2.3
PFA	2.2	63.7	25	4.9	0.6	−
OPC40C	65.8	20.7	5.8	2.3	0.3	−
CPC30R [70]	62.2	17	3.9	2.4	0.6	0.1

**Table 2:** Physical properties of aggregates used as curing agents.

Material	Density ASTM C29-17 (kg/m^3^)	Blaine ASTM C204-17 (%)	Absorption ASTM C128-15 (%)	Volumetric Weight Dry ASTM C138-17 (kg/m^3^)
Clay aggregate (EC) [60]	1840	4.1	27	990
Pumice stone (PS)	1470	4.9	30	460

**Table 3:** Description of concrete mixtures.

**Stage 1 [60] Sample Code**	**Description of Concrete Mixture**
R	Reference concrete elaborated with CPC30R (maximum consumption of cement 400 kg/m^3^).
RFA	Substitutions of CPC30R by the PFA in 15% of the mass.
IC	Internal curing with substitutions of fine aggregate by the PS (20% mass).
ICFA	Internal curing with substitutions of fine aggregate by the PS (20% in mass) and substitutions of CPC30R by the PFA in 15% of the mass.
ECFA	Substitution of coarse aggregates for clay aggregates and substitutions of CPC30R by the PFA in 15% of the mass.
**Stage 2 Sample Code**	**Description of Concrete Mixture**
R1	Reference 1 is elaborated with OPC40C (maximum consumption of the cement 400 kg/m^3^).
IC2	Substitutions of fine aggregate by the PS (20% in mass).
ICSRA	Substitutions of fine aggregate by the PS (20% in mass) with shrinkage reducing admixture.
V	Substitutions of fine aggregate by the PS (20% in mass) saturated with shrinkage reducing admixture into pores.
VFA	Substitutions of fine aggregate by the PS 20% (in mass) saturated with shrinkage reducing admixture into pores with substitutions of OPC40C by the PFA in 15% of the mass.

**Table 4:** Mixtures of concrete elaborated with CPC 30R (kg/m^3^). Stage 1 considering reference [60].

Mixture	CPC30R	PFA	SRA	PS	EC	Water (w/c = 0.35)
R	619	−	4	1440	−	214.6
RFA	510	90	4	1440	−	209.2
IC	620	−	4	1155	−	214.6
ICFA	510	90	4	1155	−	209.2
ECFA	510	90	4	1155	200	209.2

**Table 5:** Mixtures of concretes elaborated with OPC 40C (kg/m^3^). Stage 2.

Mixture	OPC 40C	PFA	FAg	CA	SRA	PS	Water (w/c = 0.40)
R1	382.5	−	889.9	712.9	2	−	153.7
IC2	382.5	−	703.5	711.7	−	130.8	138.4
ICSRA	382.5	−	699.3	707.5	2	130.1	136.3
V	403.7	−	933.3	752.7	2	112.0	162.2
VFA	323	59.5	742.9	747.1	2	112.0	165.5

**Table 6:** Volumetric weight values of all experimental concrete.

Mixtures (Stage 1) [60]	Volumetric Weight (kg/m^3^)	Mixtures (Stage 2)	Volumetric Weight (kg/m^3^)
R	2350	R1	2400
RFA	2310	IC2	2310
IC	2130	ICSRA	2310
ICFA	2160	V	2290
ECFA	2210	VFA	2260

**Table 7:** The air content and slump in the experimental structural concrete.

Mixtures (Stage 1) [60]	ASTM C185-19 [84] (%)	Mixtures (Stage 2)	ASTM C173-16 [79] (%)	Slump ASTM C143-15 [80] (cm)
R	1.5	R1	2.7	13.5
RFA	2	IC2	2.2	13
IC	1.5	ICSRA	2.2	14.5
ICFA	4	V	2.5	16.5
ECFA	3.5	VFA	2.5	21
